# ER stress is not elevated in the 5XFAD mouse model of Alzheimer's disease

**DOI:** 10.1074/jbc.RA118.005769

**Published:** 2018-10-12

**Authors:** Katherine R. Sadleir, Jelena Popovic, Robert Vassar

**Affiliations:** From the Department of Neurology, Feinberg School of Medicine, Northwestern University, Chicago, Illinois 60611

**Keywords:** amyloid, amyloid precursor protein (APP), endoplasmic reticulum stress (ER stress), Alzheimer's disease, amyloid β (Aβ), transgenic mice, neurodegeneration, protein aggregation, 5XFAD, mouse model

## Abstract

Alzheimer's disease mouse models that overexpress amyloid precursor protein (APP) and presenilin 1 (PS1) form β-amyloid (Aβ) plaques, a hallmark Alzheimer's disease lesion. It has been assumed that the neuroinflammation, synaptic dysfunction, neurodegeneration, and cognitive impairment observed in these mice are caused by cerebral Aβ accumulation. However, it is also possible that accumulation of the overexpressed transmembrane proteins APP and PS1 in the endoplasmic reticulum (ER) triggers chronic ER stress and activation of the unfolded protein response (UPR). The 5XFAD mouse, a widely used amyloid pathology model, overexpresses APP and PS1, displays aggressive amyloid pathology, and has been reported to exhibit ER stress. To systematically evaluate whether 5XFAD mice have increased ER stress, here we used biochemical approaches to assess a comprehensive panel of UPR markers. We report that APP and PS1 levels are 1.8- and 1.5-fold, respectively, of those in 5XFAD compared with nontransgenic brains, indicating that transgenes are not massively overexpressed in 5XFAD mice. Using immunoblotting, we quantified UPR protein levels in nontransgenic, 5XFAD, and 5XFAD;BACE1^−/−^ mice at 4, 6, and 9 months of age. Importantly, we did not observe elevation of the ER stress markers p-eIF2α, ATF4, CHOP, p-IRE1α, or BiP at any age in 5XFAD or 5XFAD;BACE1^−/−^ compared with nontransgenic mice. Despite lacking Aβ generation, 5XFAD;BACE1^−/−^ mice still expressed APP and PS1 transgenes, indicating that their overexpression does not cause ER stress. These results reveal the absence of ER stress in 5XFAD mice, suggesting that artifactual phenotypes associated with overexpression-induced ER stress are not a concern in this model.

## Introduction

Alzheimer's disease (AD)^2^ is characterized by the pathological misfolding and aggregation of two proteins, β-amyloid (Aβ) and hyperphosphorylated tau, which form the hallmark lesions, amyloid plaques, and neurofibrillary tangles of the disorder (1, 2). Aβ is generated by the sequential cleavage of amyloid precursor protein (APP) by two proteases. The β-secretase, β-site APP-cleaving enzyme 1 (BACE1) cuts first, releasing the extracellular domain of APP (sAPPβ) and creating a membrane-bound C-terminal fragment (β-CTF). To produce Aβ, the β-CTF is then processed by γ-secretase, which consists of four proteins (presenilin, nicastrin, Aph1, and Pen2), with presenilin 1 (PS1) providing the catalytic activity. Because mice do not naturally develop amyloid pathology, multiple mouse models that overexpress various forms of APP and PS1 with mutations that cause autosomal dominant AD (ADAD) have been developed to study amyloidogenesis in tractable animal model systems. However, it has been observed that interventions that ameliorate pathology and memory dysfunction in AD model mice have so far failed to have beneficial effects in humans, causing researchers to re-examine their mouse models with the goal of generating animal models of amyloid pathology with improved translational potential for exploring mechanisms of AD and testing novel therapeutic approaches (3). For these reasons, humanized APP knockin mice harboring ADAD mutations have been generated that do not overexpress transgenes (4).

The 5XFAD mouse is one of the most widely used models of amyloid pathology that overexpress transgenes (5). 5XFAD mice express human APP(695) with three ADAD mutations, Swedish (APP K670N/M671L) (6), Florida (I716V) (7), and London (V717I) (8), and human PS1 with two ADAD mutations (M146L and L286V) via co-integrated transgenes under the control of the neuron-specific Thy-1 promoter (5). The Swedish mutation increases BACE1 cleavage of APP, whereas the Florida and London mutations increase production of the highly amyloidogenic Aβ42 peptide compared with the less aggregation-prone Aβ40 (7, 8). The PS1 mutations increase the Aβ42:Aβ40 ratio in an additive fashion (9), further enhancing amyloidogenesis. The 5XFAD transgenes begin to express around 1 month of age, and at 1.5–2 months of age, intraneuronal Aβ is detectable. At 2–3 months, plaques have begun to form, and they increase quickly in number between 4 and 9 months of age (5), with neuronal loss evident at 9 months (10). Deficits in hippocampal memory are detected at 4–6 months (11, 12).

Because of the early onset of pathology and memory impairment, the 5XFAD model is extensively used in AD research; there are currently 391 results when the term “5XFAD” is searched in PubMed. However, concerns have been raised that endoplasmic reticulum (ER) stress caused by transgenic overexpression of the transmembrane proteins APP and PS1 may lead to ER accumulation of mis-folded proteins and activation of the unfolded protein response (UPR), which may be responsible for artifactual phenotypes in the 5XFAD model (13).

The ER is the site of folding for all membrane and secreted proteins. It contains high levels of the chaperone protein BiP/GRP78, which has both a chaperone and a stress-sensing function. Normally, BiP/GRP78 helps fold new proteins in the ER and interacts with the ER stress sensors PERK, IRE1, and ATF6, preventing their activation. When the level of unfolded proteins becomes too high, BiP releases these sensors, which then dimerize (PERK, IRE1) or translocate to the Golgi (ATF6). PERK and IRE1 autophosphorylate, resulting in up-regulation of the transcription factors ATF4, CHOP, and XBP1s, which induce the expression of genes involved in ER-associated degradation, antioxidant responses, amino acid metabolism, and apoptosis. ATF6 is cleaved, releasing ATF6f, which translocates to the nucleus and activates increased transcription of chaperones to help manage the unfolded protein burden (reviewed in Ref. 14). In the short term, these responses help the cell cope with the stress that led to the buildup of unfolded protein. However, when the insult persists and causes chronic ER stress, apoptosis can result, which could be associated with neuronal and synaptic loss in AD. It has been hypothesized that increased activation of the UPR plays a role in the development or progression of AD (reviewed by Gerakis and Hetz (52)). There are some reports suggesting that the PERK/eIF2α pathway of the UPR is activated in 5XFAD mice (15–18), but other reports suggest that it is unchanged (19).

Recently, a thorough analysis of the UPR in the APP knockin mouse model, an APP transgenic model, and P301S-Tau transgenic mice showed no elevation of ER stress (20). This study did not assess the 5XFAD mouse, so here we performed a full characterization of ER stress in the 5XFAD model. We used immunoblotting to analyze changes in levels of the proteins of the UPR in 5XFAD, nontransgenic, and 5XFAD; BACE1^−/−^ mice to determine whether any indications of ER stress were present and, if so, whether Aβ accumulation or the overexpression of the membrane proteins APP and PS1 was responsible. In sum, we did not see any changes in the UPR at any age in 5XFAD mice compared with nontransgenic or 5XFAD;BACE1^−/−^ mice, indicating that 5XFAD transgene overexpression does not cause detectable ER stress, nor do the high levels of Aβ42 and amyloid plaques observed in these mice. Our study further confirms reports that APP and APP/PS1 transgenic mice do not exhibit the UPR and, moreover, questions whether amyloid pathology causes ER stress in AD.

## Results

### Expression levels of transgenic APP and PS1 in 5XFAD mice

**Figure 1.**
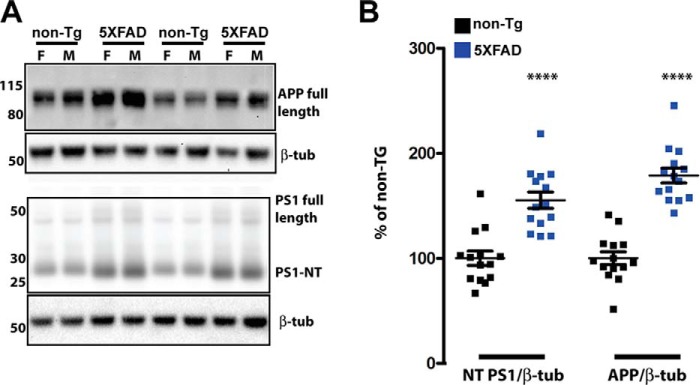
**APP and the PS1 N-terminal fragment are increased 0.8- and 0.5-fold compared with the control in 5XFAD mice at 4 months of age.** *A*, representative immunoblots of APP and PS1 in brain homogenates of 5XFAD and non-Tg mice. 20 μg of brain homogenate from non-Tg and 5XFAD mice was separated on a 4–12% BisTris gradient gel, transferred to PVDF, and then incubated with APP or PS1 antibodies that recognize both mouse and human proteins (Table 1). The *numbers* on *left* represent molecular mass in kilodaltons. *F*, female; *M*, male; β*-tub*, β3 tubulin. *B*, quantification of APP and PS1 immunoblot signals described in *A*, normalized to β3 tubulin and expressed as percentage of the non-Tg control. In 5XFAD mice, total APP is 1.8-fold of total APP in nontransgenic mice, whereas the total PS1 N-terminal fragment is 1.5-fold of that in nontransgenic controls (two-tailed Student's *t* test, *p* < 0.0001). *n* = 9 females and 7 males of each genotype; ****, *p* < 0.0001 (two-tailed Student's *t* test); *error bars* = S.E.

The levels of APP and PS1 overexpression vary between amyloid mouse models because of different promoters used and local effects of genomic insertion sites, so we started by quantifying the expression of transgenic APP and PS1 relative to the endogenous mouse proteins. Using cohorts of nine female and seven male mice each for nontransgenic and 5XFAD mice at four months of age, we quantified transgenic protein expression by immunoblot (a representative blot is shown in Fig. 1*A*). To detect APP, we used an antibody directed against amino acids 750–770 of human APP (Abcam, ab32136, rabbit monoclonal Y188, Table 1). These amino acids are 100% conserved between mouse and human, so we assume equal affinity of Y188 to APP of both species. Using this antibody, in 5XFAD mice, the total APP level was 180% of the nontransgenic APP level when results from both sexes were combined (Fig. 1*B*). As we reported previously (21), we observed that total APP was higher in 5XFAD females than in 5XFAD males, although in this cohort, the difference did not reach statistical significance (data not shown). Immunoblots of APP at 6 and 9 months were also performed (Fig. 4), and it was observed that, similar to the 4-month time point, in 5XFAD mice, the total APP level was 150–160% of the nontransgenic APP level when results from both sexes were combined, indicating that transgene expression levels remain relatively constant with age. At both time points, females again had higher APP than males, but this was not statistically significant.

**Table 1:** **Antibodies and conditions used for immunoblotting** mc =monoclonal, pc = polyclonal.

Protein	Size	Antibody name	Source	Catalog no.	Species, clonality	Concentration	Develop with	Block with
	*kDa*							
APP	110	Y188	Abcam (Epitomics)	ab32136	Rabbit, mc	1:10,000	ECL	5% milk
BACE1	70	3D5	R. Vassar		Mouse, mc	1:1,000	ECL+	5% milk
BACE1	70	D10E5	Cell Signaling	5606	Rabbit, mc	1:1,000	ECL+	5% milk
PS1-NTF	27	PS1 NT	Gopal Thinakaran		Rabbit, mc	1:2,000	ECL+	5% milk
β-Tubulin	50	TuJ1	Lester Binder		Mouse, mc	1:10,000	ECL	5% milk
ATF4	50	D4B8	Cell Signaling	11815	Rabbit, mc	1:1,000	West Femto	Pierce Superblock
p-eIF2α	38	E90	Abcam (Epitomics)	ab32157	Rabbit, mc	1:2,000	ECL+	5% milk
tot eIF2α	38	L57A5	Cell Signaling	2103	Mouse, mc	1:2,000	ECL+	5% milk
CHOP	27	D46F1	Cell Signaling	5554	Rabbit, mc	1:1,000	West Femto	Pierce Superblock
BIP	78		BD Biosciences	610978	Mouse, mc	1:2,000	ECL+	5% milk
p-IRE1-α	115		Abcam	ab48187	Rabbit, pc	1:2,000	ECL+	5% milk
tot IRE1-α	115	14C10	Cell Signaling	3294	Rabbit, mc	1:2,000	ECL+	5% milk

To measure PS1 by immunoblot, we used a rabbit polyclonal antibody directed against amino acids 1–65 of human PS1 (22). This region is 73% identical and 92% similar between mouse and human PS1, so it is possible that this antibody is more sensitive in the detection of human than mouse PS1. Most of PS1 is quickly processed to C-terminal and N-terminal fragments, which oligomerize with other subunits to form the γ-secretase enzyme. Immunoblot quantification revealed that the PS1 N-terminal fragment in 5XFAD was 150% of the nontransgenic level (Fig. 1*B*). In general, these represent relatively low levels of transgene overexpression compared with other amyloid pathology mouse models such as PDAPP, Tg2576, TgCRND8, and APP23, in which transgenic APP is overexpressed 10-fold (23), 5-fold (24), 5-fold (25), and 7-fold (26), respectively, of endogenous APP in nontransgenic controls.

### Evaluating ER stress in 5XFAD mice at 4 months of age

**Figure 2.**
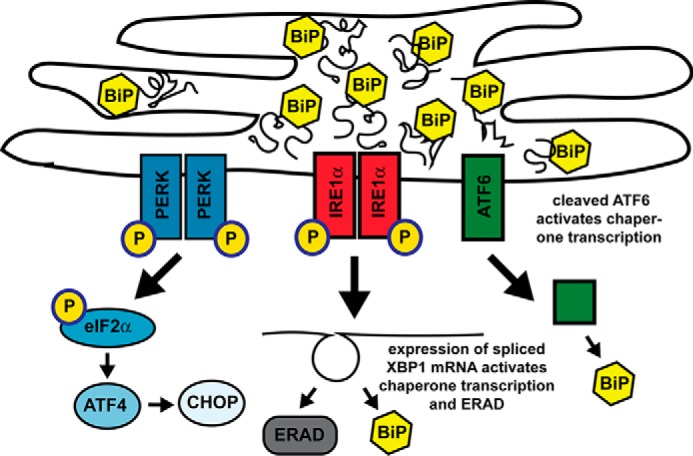
**Three arms of ER stress.** Shown is a schematic of the ER under conditions of high folding stress, when BiP is fully occupied with folded or misfolded proteins and cannot bind the ER stress sensors PERK, IRE1, and ATF6, causing them to activate the three arms of the UPR. In the first arm, p-PERK phosphorylates eIF2α, decreasing global translation but activating translation of the transcriptional activator ATF4, which induces the transcription factors CHOP and XBP1 and expression of the genes for chaperones, antioxidants, and apoptosis factors. In the second arm, p-IRE1 splices XBP1 mRNA to generate the transcription factor XBP1, which up-regulates the expression of genes for chaperones, lipogenesis, and ER-associated protein degeneration (*ERAD*) components. In the third arm, ATF6 translocates to the Golgi, where it is cleaved, generating transcription factor ATF6(N), which activates expression of chaperone and XBP1 genes.

**Figure 3.**
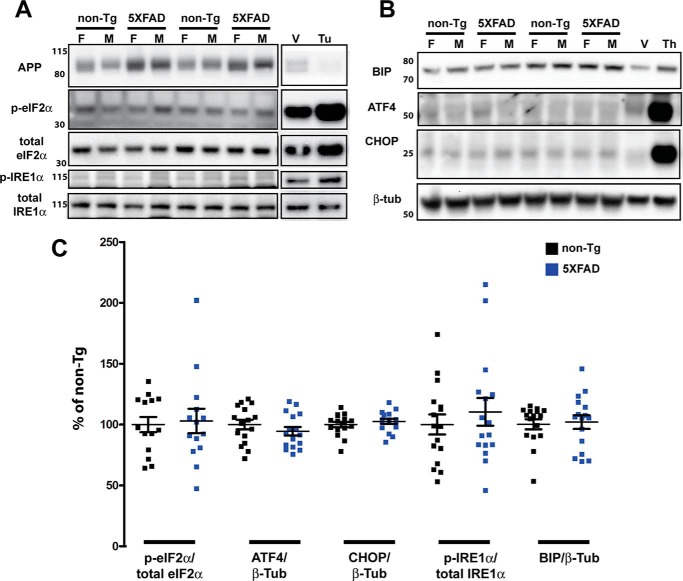
**No elevation of UPR at 4 months in 5XFAD compared with nontransgenic mice.** *A* and *B*, representative immunoblots of 30 μg of brain homogenate (APP, p-eIF2α, and total eIF2α) or 50 μg of brain homogenate (p-IRE1α, total IRE1α, BIP, ATF4, and CHOP) from 4-month-old non-Tg and 5XFAD mice separated on a 4–12% BisTris gradient gel, transferred to PVDF, and then incubated with antibodies recognizing the indicated proteins. To validate the antibodies used to detect induction of the UPR, immunoblots included 10 μg (*A*) or 20 μg (*B*) of lysate from murine primary cortical neurons treated with 5 μg/ml tunicamycin (*tu*) or DMSO vehicle (*V*) for 5 h (*A*) or 1 μm thapsigargin (*Th*) or DMSO vehicle for 24 h (*B*), in which Bip, CHOP, ATF4, and the phosphorylated forms of eIF2α and IRE1α were increased as expected. APP, which has a short *t*_½_ (51), is decreased by tunicamycin because of inhibition of global translation (*A*). Note that brains from either 5XFAD or non-Tg mice have low levels of UPR induction compared with vehicle-treated primary neurons. The *numbers* on the *left* represent molecular mass in kilodaltons. *F*, female; *M*, male. *C*, quantification of the protein immunoblot signals described in *A* and *B*, normalized to the indicated control proteins and expressed as percent of the 6-month-old non-Tg level. By two-tailed Student's *t* test, there is no significant difference between nontransgenic and 5XFAD mice (*n* = 9 females and 7 males of each genotype) for any protein analyzed. *Error bars* = S.E.

To begin our evaluation of ER stress in 5XFAD mice, we analyzed 4-month-old mice, which have a moderate amount of amyloid pathology and have been expressing 5XFAD transgenes for about 2.5 months. Before starting the study, we validated a panel of antibodies, shown in Table 1 (recognizing p-eIF2α, total eIF2α, ATF4, CHOP, p-IRE1α, total IRE1α, and BIP), that interrogate the three arms of the UPR (Fig. 2). We generated positive controls by treating primary murine neurons cultured for 7 days *in vitro* with the ER stress inducers tunicamycin (Fig. 3*A*) or thapsigargin (Fig. 3*B*). By observing an increase in ER stress proteins in the lysates of drug-treated *versus* vehicle-treated neurons, we were able to verify that our antibodies recognized the correct ER stress proteins. Additionally, by comparing the size of the increased protein band in treated neurons with that in brain homogenates, we were able to validate that we were quantifying the correct proteins *in vivo*, as some proteins such as ATF4 and p-IRE1α are in low abundance in brain homogenates, and some antibodies are not entirely specific.

Using the previously mentioned cohort of 4-month-old mice, we performed immunoblot analysis to determine the relative levels of ER stress proteins for the assessment of UPR activation in 5XFAD mice compared with nontransgenic littermates (representative blots are shown in Fig. 3, *A* and *B*) and observed no significant differences between the two genotypes for the ER stress proteins examined (Fig. 3*C*). For quantification, the phosphorylated proteins p-eIF2α and p-IRE1α were normalized to their respective total proteins, but all other proteins were normalized to β-tubulin. APP has been shown to confirm genotype and expression of the 5XFAD transgene. There was some animal-to-animal variation, as expected, especially for phospho-proteins, but no statistically significant differences in the means by two-tailed Student's *t* test were observed.

### Evaluating ER stress in 5XFAD with advanced amyloid pathology at 6 and 9 months of age

**Figure 4.**
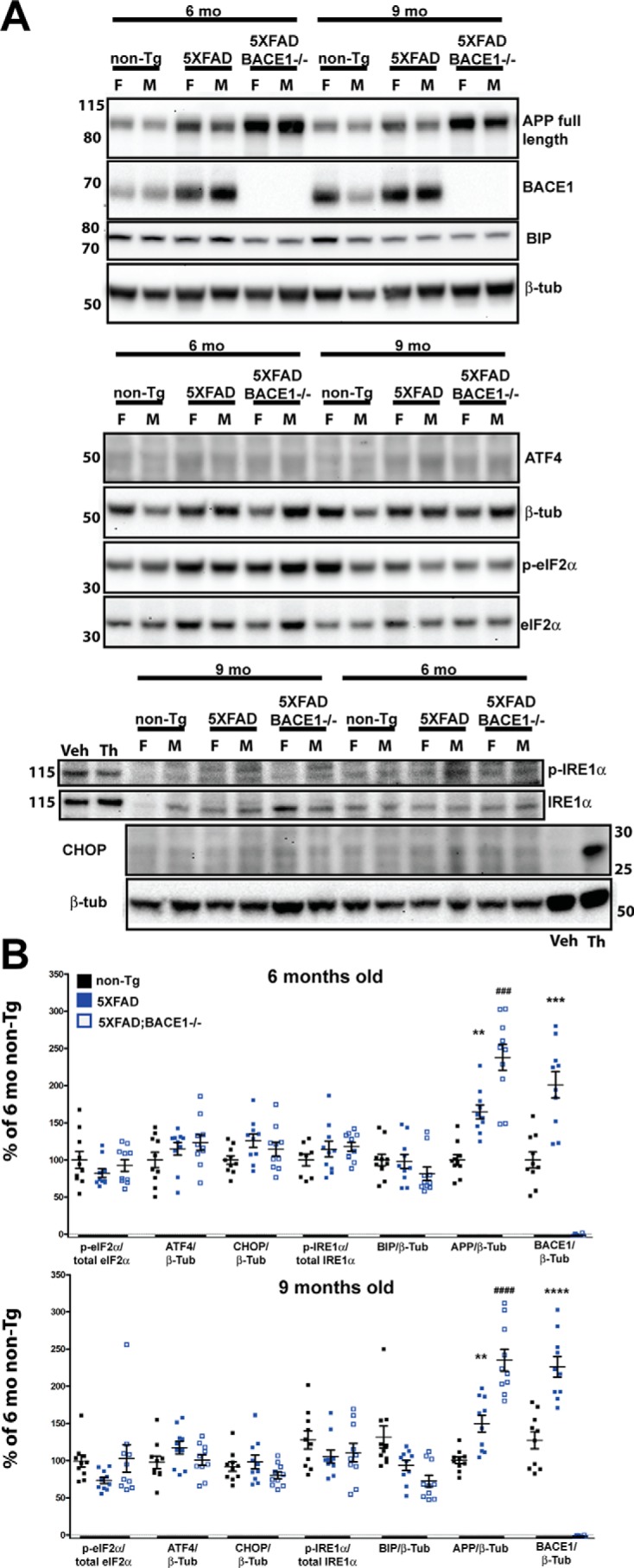
**No elevation of UPR at 6 and 9 months in 5XFAD compared with nontransgenic mice.** *A*, representative immunoblots showing 30 μg of brain homogenate (*top* and *center panels*) or 50 μg of brain homogenate (*bottom panel*) from non-Tg, 5XFAD, and 5XFAD;BACE1^−/−^ mice for each age (6 and 9 months) was separated on a 4–12% BisTris gradient gel, transferred to PVDF, and then incubated with antibodies recognizing the indicated proteins. 20 μg of lysate from murine primary cortical neurons treated with 1 μm thapsigargin (*Th*) or DMSO vehicle (*Veh*) for 24 h was included in the *bottom blot* as a positive control for p-IRE1α and CHOP. Immunoblot analysis of APP and BACE1 was included to confirm genotyping. The *numbers* on the *left* represent molecular mass in kilodaltons. *F*, female; *M*, male. *B*, quantification of the immunoblot signals described in *A*, normalized to the indicated control proteins and expressed as percent of the 6-month-old non-Tg level. By ANOVA, there is no significant difference in the levels of any UPR proteins between 5XFAD, 5XFAD;BACE1^−/−^, and nontransgenic mice (*n* = 5 females and 5 males of each genotype). In comparison, APP is significantly elevated in 5XFAD mice compared with nontransgenic mice as expected (*) and in 5XFAD;BACE1^−/−^ mice compared with 5XFAD mice (#) because of the loss of BACE1 processing. BACE1 is also significantly elevated in 5XFAD mice compared with nontransgenic mice (*) as expected (28). Note that BACE1 is undetectable in 5XFAD;BACE1^−/−^ mice. These results confirm that our immunoblotting technique can detect significant differences of more than or approximately equal to 1.5-fold. **, *p* < 0.01; *** and ###, *p* < 0.001; **** and ####, *p* < 0.0001. *Error bars* = S.E.

It is possible that prolonged exposure of the brain to overexpressed transgenic membrane proteins or to amyloid pathology may induce the UPR in 5XFAD mice. To assess this possibility, we measured the same ER stress proteins in cohorts of nontransgenic, 5XFAD, and 5XFAD;BACE1^−/−^ mice at 6 and 9 months of age (*n* = 5 per sex, per genotype, and per age). 5XFAD;BACE1^−/−^ mice, which overexpress APP and PS1 transgenes but do not generate Aβ, allowed us to determine if changes in ER stress occurred, and if so, whether they were caused by amyloid pathology or by prolonged overexpression of APP and PS1. Importantly, we observed no changes in the levels of the ER stress proteins examined at either 6 or 9 months of age in 5XFAD compared with nontransgenic mice (Fig. 4). Although total full-length APP in 5XFAD mice was ∼1.5-fold the level found in nontransgenic mice at both 6 and 9 months because of transgene expression and increased to almost 2.5-fold in 5XFAD;BACE1^−/−^ mice because of BACE1 deficiency, there was no significant difference by ANOVA in ER stress proteins in 5XFAD or 5XFAD;BACE1^−/−^ compared with nontransgenic mice at either age. At 6 and 9 months, as at 4 months, there was a nonsignificant trend toward higher APP in female compared with male 5XFAD mice (data not shown). To verify the BACE1 genotype of the mice, we performed BACE1 immunoblots and observed that the enzyme is elevated ∼2-fold at both ages, as reported previously (19, 27, 28). These results suggest that our methodology is sensitive enough to detect changes on the order of 1.5-fold differences, although it is possible that more subtle changes might be missed by immunoblot analysis of hemibrain homogenates.

**Figure 5.**
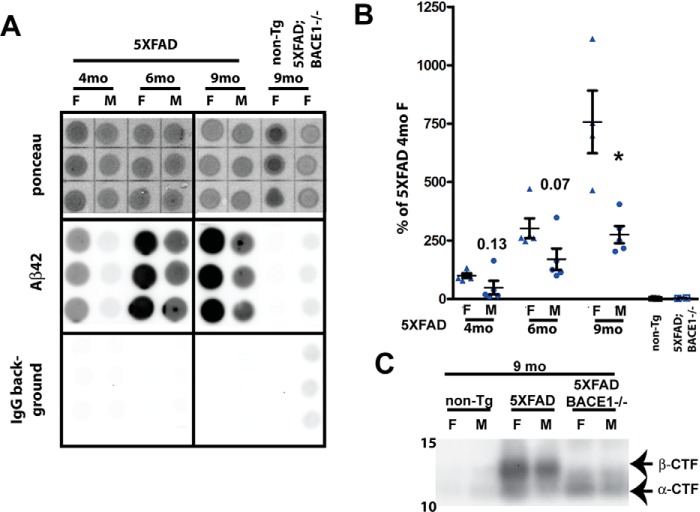
**Aβ42 and APP β-CTF are elevated in aged 5XFAD mice but do not induce UPR.** *A*, representative dot blots showing Ponceau staining, the Aβ42 immunosignal, and the IgG secondary background of brain homogenates from 5XFAD (6 and 9 months old) and negative control non-Tg and 5XFAD;BACE1^−/−^ (9 months old) mice extracted in guanidine and 3.9 μg of protein spotted in triplicate on gridded nitrocellulose membranes. Two identical membranes were made. One was incubated with anti-Aβ42 antibody followed by anti-rabbit IgG–horseradish peroxidase secondary antibody, and the other was incubated with secondary antibody only (IgG background). *F*, female; *M*, male. *B*, quantification of Aβ42 dot blot signals described in *A*. The IgG secondary antibody background was subtracted from the Aβ42 signal, which was then normalized to Ponceau stain intensity and expressed as percentage of the female 5XFAD level at 4 months of age. Aβ42 levels increase over time from 4 to 9 months but, in males, are only ∼40–60% of those in females at a given age, although the difference is significant only at 9 months because of large variation between mice. Numbers represent *p* values at 4 and 6 months; *, *p* < 0.05 at 9 months (two-tailed Student's *t* test). *n* = 5 females and 5 males for each genotype. *Error bars* = S.E. *C*, immunoblot analysis of homogenates from representative brains of 9-month-old mice using antibody Y188 recognizing the C terminus of APP confirms that APP β-CTF, although barely detectable in nontransgenic mice, is elevated in 5XFAD mice and absent from 5XFAD;BACE1^−/−^ mice, which still have notable amounts of APP α-CTF because of α-secretase cleavage.

To verify the levels of Aβ accumulation in these cohorts of mice, we performed dot-blot analysis on guanidine HCl–extracted brain homogenates using an Aβ42-specific antibody (Fig. 5, *A* and *B*). This method has been validated as an effective and inexpensive approach to determine relative Aβ levels in transgenic mice and human brains (21, 29). Our cohorts of mice were not perfused at harvest, so some brain homogenates had a high background with secondary antibody alone, presumably because of high immunoglobulin contamination from blood in the sample. Therefore, we subtracted the signal of the secondary antibody alone from the Aβ42 signal before normalizing to Ponceau stain intensity (21). As reported previously, Aβ42 levels increased dramatically between 4 and 9 months of age (5). There was a trend for higher Aβ42 in female 5XFAD mice, although this became significant only at the 9-month time point. Our previous work (21) reported significant differences between females and males in Aβ42 quantified by dot-blot at 4, 6, and 9 months, but larger sample sizes were used, *n* = 7–17, compared with *n* = 5 in this study. Because the magnitude of the difference is similar in the two studies, with male 5XFAD mice having consistently 40–60% of the Aβ42 level as females, the lack of statistical significance likely reflects the smaller sample size and the high inter-animal variation in amyloid pathology, even within the same sex.

Using our APP C-terminal–specific antibody, we performed an immunoblot analysis for the APP β-CTF, confirming that it is significantly elevated in 5XFAD mice (Fig. 5*C*). The α- and β-CTFs are barely detectable under these conditions in nontransgenic mice, preventing relative quantification, but the APP β-CTF is the most prominent band in 5XFAD brain homogenates, whereas it is absent in 5XFAD;BACE1^−/−^ mice. Our previous work has shown that, in 5XFAD mice, the β-CTF, like APP transgene expression and Aβ42, was elevated in females compared with males (21) but did not address the role it may play in the phenotypes of the 5XFAD mouse. The 5XFAD;BACE1^−/−^ mice are informative controls for differentiating the effects of APP and PS1 overexpression *versus* amyloid pathology. However, because both Aβ and β-CTF are absent in 5XFAD;BACE1^−/−^ mice, the effects of these two substances cannot be easily separated in this model. In sum, our results show that, despite prolonged exposure to overexpressed transgenic APP and PS1 and high levels of Aβ42, there is no evidence of ER stress leading to activation of the UPR in the 5XFAD mouse model of amyloidosis.

## Discussion

Here we assess the possibility that, in the 5XFAD mouse model, overexpression of APP and PS1 transgenes leads to ER stress and activation of the UPR and that this could be the cause of phenotypes attributed to high levels of amyloid pathology (13). In sum, we did not find evidence that ER stress protein levels were elevated in the brains of 5XFAD mice at 4, 6, or 9 months of age despite constant levels of transgene expression and rapidly increasing cerebral Aβ42 accumulation. Although we did detect increased amyloid in female 5XFAD mice compared with males, we saw no differences between the sexes in the levels of ER stress response proteins (data not shown), further supporting the hypothesis that amyloid deposition is not driving ER stress. This is in agreement with several other recent publications that do not find ER stress in APP/PS1 double-transgenic mouse models of amyloidosis (19, 20, 30, 31). Concerns were raised about ER stress especially in these double-transgenic mice because, in addition to overloading the ER with overexpressed membrane proteins, reports showed that ADAD PS1 mutations caused changes in calcium handling and homeostasis (32–34). Because the ER plays a key role as a calcium reservoir, altered calcium handling could cause ER stress and induction of the UPR, but the data presented in the current report indicate that the ADAD mutations in PS1 do not lead to ER stress in the 5XFAD mouse model.

Although we found no evidence of ER stress in 5XFAD mice, the possibility exists that ER stress is transiently induced upon initial expression of the APP and PS1 transgenes around 1–1.5 months of age and persists until homeostatic mechanisms rebalance ER chaperones to handle the increased protein folding load and bring ER stress levels back down to normal. Future experiments to analyze ER stress protein levels before, during, and after the start of transgene expression in 5XFAD brain will be required to answer this question.

Although more and more evidence is accumulating that high levels of Aβ do not induce the UPR in APP transgenic mice, there are histological data from human AD patients that do support the hypothesis that the UPR is activated in some neurons in human AD and in other neurodegenerative disorders (reviewed in Ref. 35). In studies of AD and tauopathy brains (progressive supranuclear palsy, hereditary frontal temporal dementia with parkinsonism linked to chromosome 17, corticobasal degeneration), increased p-PERK, p-eIF2α, and p-IRE1 are observed compared with controls (36–42). In most of these reports, markers of the activated UPR are observed to be associated with abnormally phosphorylated tau and/or granulovacuolar degeneration (GVD) in regions of the brain affected by disease and are not localized near amyloid plaques (reviewed in Ref. 35). This is supported by a histological comparison of APP/PS1 mice and pR5 mice, which express hTau40 with the P301L mutation, that found increased GVD, p-PERK, and p-eIF2α with age only in the pR5 tauopathy model mice and primarily in neurons that have abnormally phosphorylated pre-tangle tau (31). Using P301L tau transgenic mice that express a shorter form of human Tau (43), Ho *et al.* (44) observed elevated p-PERK and p-eIF2α localized with tau phosphorylated at Thr-231 and Ser-235 in the hippocampus of aged transgenic but not control mice (44). Using primary neurons, they demonstrated that ER stress can induce tau phosphorylation and vice versa, suggesting the possibility of a vicious cycle in AD, where abnormal tau phosphorylation leads to ER stress and increased tau pathology (44). However, these observations of ER stress may be specific to certain Tau mutations or Tau transgenic mouse lines, as recent work shows no evidence of ER stress in the 3XTg mouse, which has both Tau and APP transgenes, or a Tau P301S line (20).

Taken together, these data suggest that ER stress may be up-regulated in human AD and may even play a role in neuronal degeneration, but if so, it is more likely downstream of tau pathology and not directly related to amyloid. As most APP transgenic mice do not show the tau tangle formation or increased GVD characteristic of human AD pathology or the ER stress changes seen in human disease, amyloid pathology mice may not be useful models for studying ER stress and UPR dysregulation in AD. Tauopathy models may prove to be more fruitful, although there are not yet mouse models that faithfully recapitulate the same tau pathology observed in AD.

Another concern raised with the APP-overexpressing models is the increased levels of the APP β-CTF produced by BACE1, which can be quite high, especially in mouse lines carrying the APP Swedish mutation, which increases BACE1 cleavage of APP. There is evidence that the APP β-CTF may have detrimental effects, such as inducing lysosomal and autophagic pathology (45), causing endosomal dysfunction (46), and impairing anterograde axonal transport (47). These are all features of AD pathology that have generally been attributed to elevated amyloid or abnormally phosphorylated tau, but the possibility remains that elevated β-CTF could play a role as well. On the other hand, overexpression specifically of the β-CTF did not cause neuron or synapse loss (48). Moreover, viral expression of transcription factor XBP1s in the hippocampus of CRND8 APP transgenic mice rescued spine density, synaptic plasticity, and memory function and reduced soluble Aβ levels with no effect on β-CTF levels, suggesting that Aβ, not β-CTF, is responsible for dysfunction in this mouse model (49). Further work in models such as the APP NL knockin mouse, which accumulates the same amount of β-CTF as the APP NL-F knockin mouse but does not accumulate Aβ (4), will be useful in determining whether the β-CTF alone has toxic effects.

There are other possible caveats to transgenic overexpression of proteins, such as increased activity of the overexpressed proteins, loss of endogenous expression patterns and mRNA splicing, disruption of chromosomal loci by insertion, and variable expression of the transgene (4). The use of an APP/PS1 transgenic model like the 5XFAD mouse on a BACE1^−/−^ background as a control can help separate the effects of increased Aβ and other APP products from the effects of the transgene itself. Any phenotype as a result of the transgene alone will be present in the transgenic BACE1^−/−^ mouse whereas those related to Aβ will not.

In this work, we have demonstrated that the 5XFAD mouse model of amyloid pathology, like other APP/PS1 and APP transgenic mice, does not develop ER stress because of overexpression of the 5XFAD transgenes or high levels of Aβ. This eliminates the possibility of increased ER stress leading to artifactual phenotypes, although the effects of the elevated β-CTF in these types of mouse models require further investigation to determine whether they may contribute to phenotypes generally attributed to Aβ. The lack of ER stress and UPR activation in amyloid pathology mouse models does not mean that they play no role in human AD. Indeed, human and some mouse model data suggest that they do, but in the context of tau pathology rather than amyloidosis, indicating that certain mouse models of tauopathies may be more useful in determining the role of ER stress in AD.

## Experimental procedures

### Mice

5XFAD mice were generated as described previously (5) and maintained by crossing transgene-positive males with B6/SJL F1 hybrid females (The Jackson Laboratory). As negative controls, 5XFAD mice were crossed to BACE1^−/−^ (50) mice to generate 5XFAD;BACE1^−/−^ mice. All animal work was done in accordance with Northwestern University Institutional Animal Care and Use Committee (IACUC) approval.

### Primary neurons for ER stress controls

Embryonic day 15.5–16.5 C57BL/6 mouse cortical neurons were plated on poly-l-lysine–coated 10-cm dishes (7.5 × 10^6^ cells/dish) in Neurobasal medium supplemented with 2% B-27, 500 μm glutamine, 10% horse serum, and 2.5 μm glutamate (all from Thermo Fisher). After 2 h, the medium was changed to Neurobasal with 2% B-27, 500 μm glutamine, and 2.5 μm glutamate. After 1–3 days, the medium was replaced with Neurobasal plus 2% B-27 and 500 μm glutamine. Following 7 days in culture, neurons were treated with 1 μm thapsigargin or DMSO vehicle for 24 h or 5 μg/ml tunicamycin or DMSO vehicle for 5 h. To collect lysates, dishes were rinsed once in PBS, lysed in 300–500 μl of radioimmune precipitation assay buffer, and spun for 10 min at 10,600 × *g* at 4 °C. The protein concentration of the supernatant was quantified using the BCA assay (Pierce), and the supernatant was used for immunoblotting.

### Immunoblotting

Snap-frozen hemibrains were homogenized in 1 ml of 1× PBS with 1% Triton X-100 supplemented with protease inhibitors (Calbiochem) and Halt phosphatase inhibitor mixture (Thermo Scientific). Protein concentration was quantified using a BCA assay (Pierce). Thirty or fifty micrograms (for ATF4, CHOP, total, and p-IRE1α) of brain homogenate and 10 or 20 μg of neuronal lysates were separated on 4–12% BisTris gels (Invitrogen NuPage), and protein was transferred to a 0.45-μm PVDF membrane. For detection of PS1 NT, samples were mixed with standard Laemmli buffer but not boiled prior to electrophoresis (22). Membranes were Ponceau S–stained immediately after transfer and then probed overnight at 4 °C or for 1 h at room temperature (TuJ1) with primary antibody under the conditions shown in Table 1. This was followed with a 1-h incubation with secondary horseradish peroxidase–conjugated anti-mouse or anti-rabbit antibody (Vector Laboratories, PI-2000 or PI-1000, 1:5000) in either milk or 5% milk or Pierce Superblock, as indicated in Table 1. Blots were visualized using the Pierce reagents ECL, ECL Plus, or SuperSignal WestFemto, and signals were imaged using a FluorChemR imager (ProteinSimple) and then quantified with Alphaview software (ProteinSimple). Signals were normalized to tubulin or, for phosphorylated proteins, to the corresponding total protein. Statistics were done as described below. For these analyses, multiple gels were cut into horizontal strips and stacked so all samples for a given protein were transferred to a single piece of PVDF membrane. Putting all samples (up to 60) on one membrane eliminated the need to account for variation in transfer, antibody incubation, and ECL application that can occur between blots.

### Dot blotting

For Aβ42 dot blots, 10 mg/ml brain homogenates were extracted in 1.56 volumes of 8.2 m guanidine hydrochloride (GuHCl) and 82 mm Tris-HCl (pH 8.0) (5 m GuHCl final) over three nights on a nutator. Then 1 μl of GuHCl-extracted sample (3.9 μg total protein) was spotted in triplicate on a gridded nitrocellulose membrane, dried for 1 h at 37 °C, rinsed in TBS-T (Tris-buffered saline with 0.1% Tween) and then water, and stained with Ponceau S. Two identical blots were made and incubated in either 1:4000 anti-Aβ42 rabbit mAb (clone H31L21, Invitrogen, 700254) or 5% milk only (primary delete), followed by horseradish peroxide–conjugated secondary antibody (Vector Laboratories, PI-1000). The duplicate blots incubated with anti-Aβ42 antibody or primary delete were developed together with ECL SuperSignal WestPico (Pierce), imaged simultaneously using a FluorChemR imager (ProteinSimple), and then quantified with Alphaview software (ProteinSimple). The signal from the primary delete blot was subtracted from the Aβ42 signal; this was normalized to Ponceau S staining and the triplicates were averaged, and statistics were performed as described below.

### Statistics

Student's two-tailed *t* test and ANOVA were done using InStat software (GraphPad Software, Inc., San Diego, CA) to compare means of the various genotypes and genders. For the scatter plots, * and # indicate groups statistically compared by Student's *t* test or ANOVA; * 0.05 > *p* > 0.01 ** 0.01 > *p* > 0.001 *** 0.001 > *p* > 0.0001. Error bars indicate standard error. * indicates comparison with the non-Tg group, whereas # indicates comparison with the 5XFAD group.

## Author contributions

K. R. S. and R. V. conceptualization; K. R. S. formal analysis; K. R. S. and J. P. investigation; K. R. S. and R. V. methodology; K. R. S. writing-original draft; K. R. S., J. P., and R. V. project administration; K. R. S. and R. V. writing-review and editing; R. V. resources; R. V. funding acquisition.

## Footnotes

2 The abbreviations used are:

AD: Alzheimer's disease
Aβ: amyloid β
APP: amyloid precursor protein
CTF: C-terminal fragment
ADAD: autosomal dominant Alzheimer's disease
ER: endoplasmic reticulum
UPR: unfolded protein response
ANOVA: analysis of variance
GVD: granulovacuolar degeneration
PVDF: polyvinylidene difluoride
GuHCl: guanidine hydrochloride
Tg: Transgenic.
